# Hemidouble Stapling Technique versus Ghosting Double Stapling Technique for Esophagojejunostomy after Laparoscopic Total Gastrectomy

**DOI:** 10.3390/jpm14030314

**Published:** 2024-03-18

**Authors:** Serdar Senol, Servet Karagul

**Affiliations:** Department of General Surgery, Surgical Gastroenterology, Samsun Training and Research Hospital, İlkadım 55090, Samsun, Turkey; servetkaragul@hotmail.com

**Keywords:** esophagojejunostomy, gastrectomy, laparoscopy, surgery

## Abstract

The hemidouble stapler technique (HDST) in laparoscopic intracorporeal esophagojejunostomy has aspects that pose risks to the safety of the anastomosis. We developed a new esophagojejunostomy technique that converts a double-stapled anastomosis to a single-stapled anastomosis in laparoscopic total gastrectomy (LTG). The aim of this study is to compare the results of two techniques. Patients who underwent LTG for gastric cancer in our hospital between October 2016 and May 2022 were included in the study. Patients were retrospectively reviewed in two groups: those who underwent HDST and the ghosting double stapling technique (GDST). Both groups were analysed in terms of demographics, perioperative findings, and postoperative outcomes. The GDST was used in 14 patients. The HDST was used on 16 patients. Two patients in the HDST group whose esophagojejunal anastomosis was not assessed on endoscopic imaging were excluded. The mean total operative times were 292.6 ± 43.7 and 224.3 ± 36.1 min (*p* < 0.001). The mean times for esophagojejunostomy were 38.6 ± 4.3 and 26.8 ± 6.4 min (*p* < 0.001). One case of anastomotic stenosis was observed in the HDST group. Anastomotic leakage was not observed in both groups. However, there was no significant difference in overall morbidity between the groups (*p* > 0.05). Both HDST and GDST can be safely performed in the esophagojejunostomy for LTG.

## 1. Introduction

Gastric cancer is the fifth most common cancer and the fourth leading cause of cancer-related death worldwide [1]. Surgery is still the only chance of a cure for gastric cancer [2]. Laparoscopic gastrectomy is used by experienced surgeons for the surgical treatment of gastric cancer. Increased experience in laparoscopic surgery is associated with improved surgical quality and prognosis in patients with gastric cancer. Technological advances have been effective in developing minimally invasive approaches, and the fundamentals of the technique, such as a negative resection margin and adequate lymphadenectomy, continue to be applied. Minimally invasive surgical approaches have been shown to be oncologically equivalent to better recovery, and surgery continues to move towards less invasive techniques for the appropriately selected patient. It is important to recognise that there is a learning curve with laparoscopic procedures, and therefore, the indication for this approach should be decided with due regard to the expertise of the surgeons and the individual assessment of each patient [3,4,5,6]. 

Laparoscopic total gastrectomy (LTG) has been shown to be a reliable surgical approach for the treatment of gastric cancer, with no increase in overall postoperative complications. It offers better cosmesis, less invasiveness and faster recovery. LTG is a safe and feasible surgical approach with short-term results for upper and middle gastric cancer. Although more high-quality, large-sample, multicentre randomised trials are needed, LTG is not inferior to open total gastrectomy in terms of oncological outcomes in advanced gastric cancer and has advantages in terms of perioperative outcomes [7,8,9,10,11,12]. However, LTG has not become as widely used as laparoscopic distal gastrectomy due to difficulties in performing esophagojejunal anastomosis [13]. 

In fact, the main issue that deterrent to performing laparoscopic total gastrectomy is the complicated esophagojejunostomy anastomosis during reconstruction. Esophagojejunostomy (EJ) anastomosis is both technically difficult and can cause problems with serious implications for patient morbidity and mortality. Failure of an EJ anastomosis leads to adverse postoperative events that have a long-term impact on the patient’s quality of life. The main difficulties are the narrow field of view and the retraction of the esophagus upwards from the hiatus. Pulling the esophageal stump downwards and attempting to maintain healthy vascularisation by moving the small bowel upwards requires experience and skill to achieve a smooth laparoscopic procedure. Surgeons have used a variety of reconstruction methods, but there is no standard method for esophagojejunostomy. To find a simple and easy-to-perform method, different reconstruction methods have been used in the literature and evaluated in terms of anastomotic leakage, anastomotic stenosis, postoperative dysphagia, reflux, operative time and cost [14,15,16,17,18,19,20,21,22,23]. Mechanical staplers are widely used for safety and time-saving. Although the technical details and procedures vary, stapled anastomoses can be divided into two main categories: circular and linear stapling techniques.

In circular stapler anastomosis, the use of purse-string sutures when attaching the anvil of the circular stapler to the esophageal stump is a challenging step in LTG due to difficulties in visualisation and manoeuvrability. This method, known as the single stapling technique (SST), uses a single circular stapler and, by avoiding the use of linear staplers, eliminates staple intersections, which are known to cause anastomotic leakage and stenosis. On the other hand, the double stapling technique (DST) facilitates reconstruction by using circular and linear staplers in laparoscopic surgery. However, in DST, the linear stapler and the circular stapler overlap in two places, resulting in the formation of risky areas that may be the cause of anastomosis-related complications.

The hemi-double stapling technique (HDST), which uses linear staples and circular staples in the anastomosis, eliminates one of the staple intersections and one of the dog ears by placing the anvil bar close to one end of the linear staple line and ensuring the excision of this portion during the anastomosis. However, the fact that an additional linear staple line, an overlapping staple area and a dog ear remain in the anastomosis after HDST still raises concerns about the safety of the anastomosis, although less so than with DST where there are two of each [24,25]. 

We described a new form of double stapling technique that does not involve dog ears or crossovers in esophagojejunostomy. Since the linear staple line was completely resected and disappeared in this novel procedure, we named it the ghosting double stapling technique (GDST). In this study, we aimed to compare the HDST with the GDST we used for esophagojejunostomy in laparoscopic total gastrectomy.

## 2. Materials and Methods

Patients who underwent LTG and esophagojejunostomy using HDST and GDST in our hospital between October 2016 and May 2022 were retrospectively analysed. 

Exclusion criteria were open procedures, surgery for diagnoses other than cancer, subtotal gastrectomy, and patients whose esophagojejunal anastomosis was not evaluated by endoscopic imaging within one year after surgery. Ethical approval was obtained from the local ethics committee (number 2022/6/5). Written informed consent for LTG was obtained from all patients. Age, sex, body mass index (BMI) and American Society of Anesthesiologists (ASA) score were recorded. Total operative time, time to esophagojejunal anastomosis, intraoperative blood loss, specimen extraction site, time to oral intake, hospital stay, postoperative complications, pathological results and survival outcomes were evaluated. 

### 2.1. Surgical Technique

**Figure 1:**
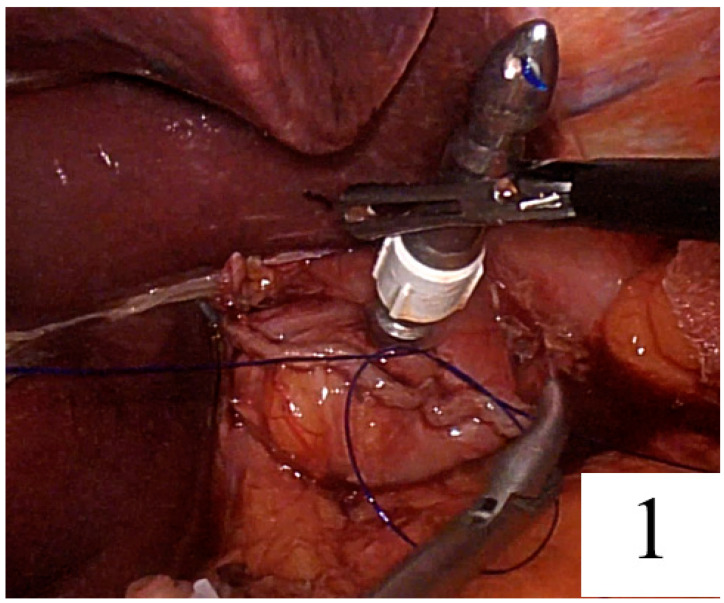
U-shaped purse suture knotting at the anterior side of the rod.

After omentectomy, devascularisation of the stomach, division of the duodenum and D2 lymphadenectomy, surgery continued to prepare the esophagus for reconstruction. In the first step of both techniques, approximately 8 cm of polypropylene suture plus needle was tied to the end of the anvil of a 25 mm circular stapler. The stomach was pulled caudally. The esophageal wall was then penetrated, and a semi-circumferential esophagotomy was performed. The anvil was inserted into the esophagus through this incision and advanced towards the proximal esophagus. The needle of the suture at the tip of the anvil was passed through the anterior esophageal wall and then withdrawn from the esophagus. The linear stapler was located perpendicular to the long axis of the esophagus and closed between the penetrated suture and the open window of the esophageal wall. All knots and the tip of the rod were extracted through the linear stapling line. The esophagus was immediately transected, and the entire rod was extracted through the staple line. In the HDST, as described by Omori et al. [25], the anastomosis was accompanied by a dog ear and an overlapping staple point by pulling the thread to the right side when clamping the esophagus with a linear stapler. In the GDST, the tip of the anvil rod was extracted through the centre of the staple line. The esophagus was then transected. We planned to remove the entire linear staple line of the esophageal stump and keep the linear staple line within the circular knife by using a laparoscopic suture when the circular stapler was closed. Therefore, a U-shaped purse-string suture was performed by passing the needle (2/0 prolene suture) through the upper right, lower middle and upper left corners of the stump, respectively, and knotting at the anterior side (Figure 1). 

**Figure 2:**
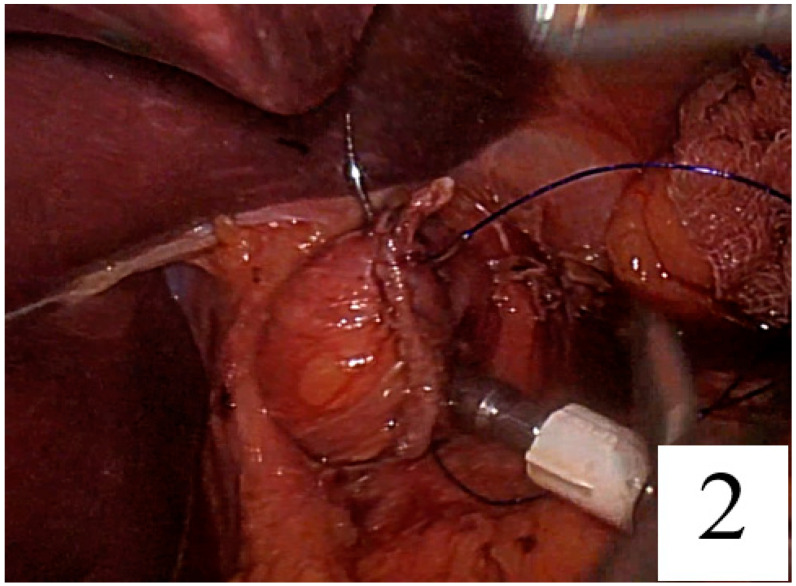
Passing through the needle from the right corner of the linear stapler line.

**Figure 3:**
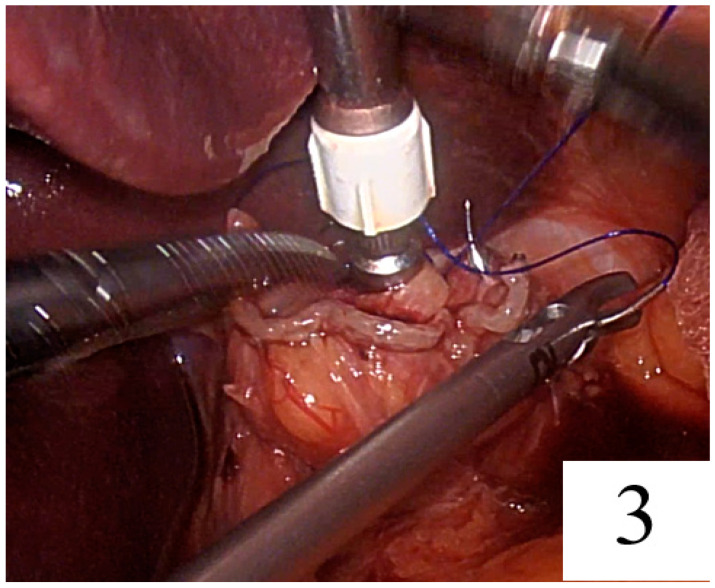
Passing through the needle from the left corner of the linear stapler line.

**Figure 4:**
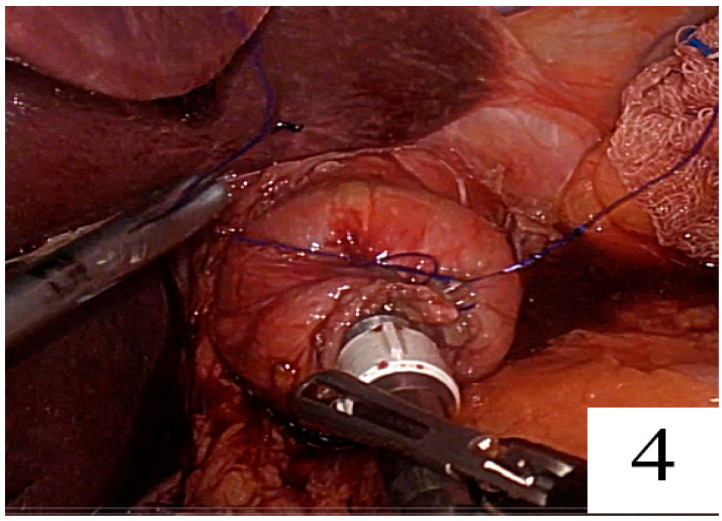
Tying the suture at the anterior side of the rod.

The needle was passed seromuscularly first from the right and then from the left corner of the linear staple line, and the suture was tied at the anterior side of the rod (Figure 2, Figure 3 and Figure 4).

**Figure 5:**
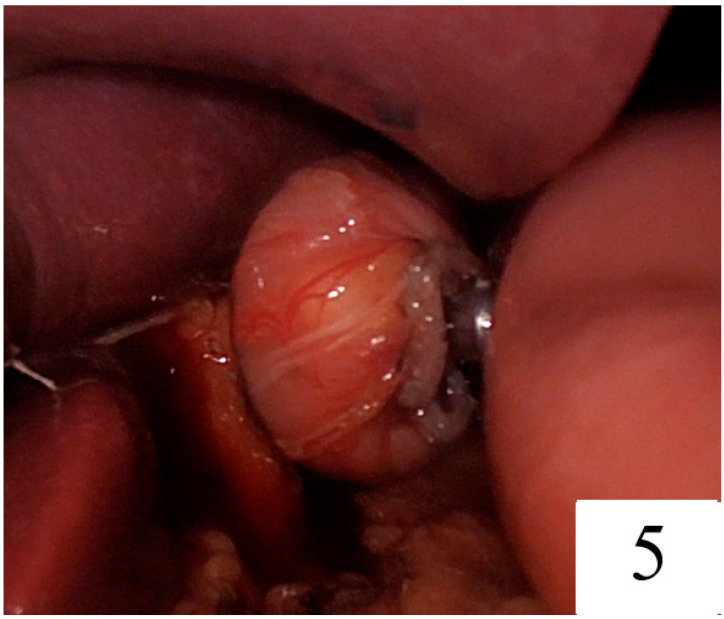
Just before firing the circular stapler, it was ensured that the linear stapler line was inside the circular knife.

The transverse colon was elevated, the ligament of Treitz’s was visualised, and the jejunum was divided approximately 25 cm distally. The 10-millimetre trocar incision on the left was enlarged to allow insertion of the circular staple. A 25 mm circular stapler was inserted into the jejunum. The esophagojejunostomy was completed by observing under the laparoscopic vision that the linear staple line remained within the circular staple line and could be completely removed (Figure 5). 

**Figure 6:**
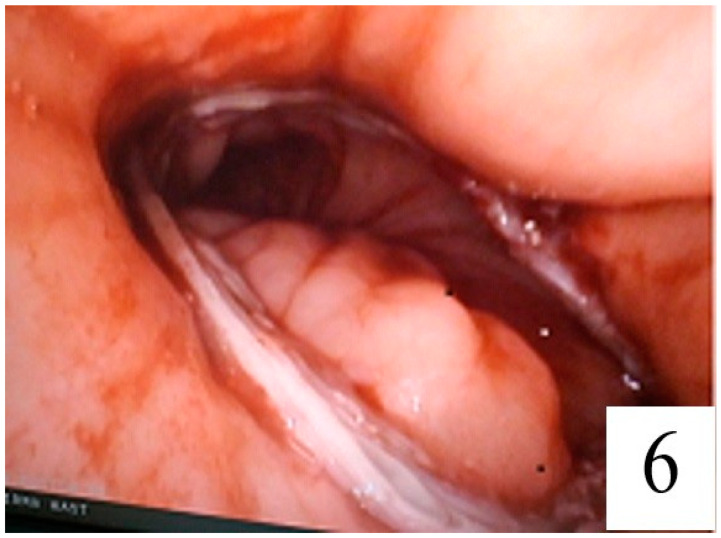
Endoscopic view of the anastomosis.

After completion of the esophagojejunostomy, the anastomosis was evaluated endoscopically (Figure 6).

In both techniques, a side-to-side jejunojejunostomy was created using a laparoscopic linear stapler approximately 40 cm distal to the esophagojejunal anastomosis. The staple orifice was closed intracorporeally with 3/0 polypropylene sutures. The specimen was removed from the abdomen via the extended trocar incision, suprapubic incision, or transvaginal route.

A nasogastric tube was not placed in any patient. Oral fluid intake was usually resumed on postoperative day 3, and a soft, solid diet was started on day 4.

### 2.2. Statistical Analysis

Statistical analyses were performed using “IBM SPSS Statistics for Windows. Version 25.0 (Statistical Package for the Social Sciences, IBM Corp., Armonk, NY, USA)”. Descriptive statistics are presented as “n” and “%” for categorical variables and mean ± SD for continuous variables. Mann–Whitney U test, a nonparametric test, was used for pairwise group comparisons, and Pearson Chi-Square test—Fisher’s Exact test was used for categorical variables. The Kaplan–Meier method was used to compare survival times between various clinical parameter groups. All *p* values < 0.05 were considered significant.

## 3. Results

Between October 2016 and May 2022, we performed 30 laparoscopic total gastrectomies for gastric cancer in our hospital. HDST was used in 16 patients. GDST was used in 14 patients. None of the patients required any further open surgeries. In the HDST group, two patients died after the surgery. One due to acute respiratory distress syndrome on postoperative day 42 and the other one due to an acute cerebrovascular event on postoperative day five. These patients whose esophagojejunal anastomosis was not evaluated on endoscopic images were excluded from the study.

Table 1 shows the patient and tumour characteristics. Sex, age, body mass index, histologic classification, disease stage and distance from the surgical margin were not significantly different between the two groups. The number of ASA III patients and patients who received neoadjuvant therapy was significantly higher in the GDST group (10 vs. 5; *p* = 0.035 and 10 vs. 5; *p* = 0.023, respectively). 

Surgical details are summarised in Table 2. Total operative time and time to perform esophagojejunal anastomosis were significantly longer in the GDST group (292.6 ± 43.7 vs. 224.3 ± 36.1 min; *p* < 0.001, and 38.6 ± 4.3 vs. 26.9 ± 6.2; *p* < 0.001, respectively.) Blood loss, conversion to open surgery, and specimen removal were not significantly different between the two groups.

The mean time to oral intake was 3.4 ± 0.6 days, and the mean time of hospitalisation was 8.2 ± 3.1 days in the HDST group. In the GDST group, the mean time to oral intake was 3.2 ± 0.5 days, and the mean time of hospitalisation was 6.5 ± 1.2 days. The differences were not statistically significant.

In the HDST group, one case of paralytic ileus was treated with electrolyte replacement and discharged. One patient developed dysphagia during the first year of postoperative follow-up. Upper endoscopy revealed an esophagojejunal anastomotic stenosis. The patient was treated with endoscopic dilatation and recovered. In the GDST group, one case of paralytic ileus was treated with electrolyte replacement. One patient developed a grade A pancreatic fistula, which was successfully treated with medical therapy. One patient developed a pulmonary embolism, which was eventually resolved with anticoagulant therapy. No post-operative mortality was observed in the GDST group. In both groups, patients were discharged without the need for interventional or surgical procedures. There were no complications related to the esophagojejunal anastomosis. However, postoperative morbidity did not differ between the two groups (*p* = 1.000).

The mean follow-up period was 15.1 ± 19.3 months and 19.1 ± 11.5 months in the HDST and GDST groups, respectively (*p* = 0.137). The number of surviving patients with less than three years was two in the GDST group and six in the HDST group. Before death, in the HDST group, peritoneal recurrence was confirmed in four patients (28.5%), and no distant metastases were confirmed. There was a case with simultaneous peritoneal recurrence and distant metastases in the GDST group. Two patients in the HDST group and one in the GDST group with no evidence of disease recurrence died due to acute cerebrovascular events, pulmonary embolism, and acute myocardial infarction, respectively. Three-year overall survival was 85% vs. 30% in the GDST and HDST groups, respectively (*p* = 0.012).

## 4. Discussion

Recently, the World Society of Emergency Surgery (WSES) expert panel reviewed the literature and suggested laparoscopy as the first approach for stable patients undergoing emergency abdominal surgery for general surgical emergencies and abdominal trauma [26]. Based on this, we believe that laparoscopy would be used much more widely in elective gastrointestinal cancer surgery, even in more complex cases. In addition, in stable patients who develop postoperative complications, initially planning a laparoscopic approach seems to be an appropriate option.

The use of laparoscopic surgery for gastric cancer has increased significantly. Laparoscopic distal gastrectomy (LDG) with D2 lymph node dissection for locally advanced gastric cancer has been shown to be non-inferior to open distal gastrectomy. Laparoscopic surgery has been found to be more advantageous in terms of intraoperative blood loss, postoperative complications and rapid postoperative recovery, even for advanced gastric cancer, and it has been stated that laparoscopic distal gastrectomy is safe and effective in the treatment of elderly patients with distal gastric cancer. Laparoscopic distal gastrectomy is considered to be oncologically sufficient in terms of lymph node dissection, adequacy of resection and survival. LDG and D2 lymph node dissection, when performed by experienced surgeons in high-volume specialised centres, is a safe and effective technique for patients with advanced gastric cancer and is increasingly replacing open distal gastrectomy. This laparoscopic approach may become the standard of care for locally advanced distal gastric cancer [27,28,29,30,31]. 

In total gastrectomy, adaptation to minimally invasive surgery has not shown the same acceleration as in distal gastrectomy. Many surgeons have been reluctant to perform LTG due to technical difficulties, particularly with anastomosis. As with open surgery, laparoscopic total gastrectomy has the potential to cause very serious complications. Serious situations such as esophagojejunostomy leak, lung infection, anastomotic bleeding, anastomotic stenosis and death can occur. For this reason, performing LTG without appropriate surgical training and sufficient laparoscopic experience may lead to unsatisfactory results [32,33,34]. In the study by Etoh et al., which is one of the most influential studies comparing laparoscopic total gastrectomy with open total gastrectomy, the incidence of anastomotic leakage was 5.3% in laparoscopic total gastrectomy (LTG) and 6.1% in open total gastrectomy (OTG). The authors found no significant difference between laparoscopic and open total gastrectomy in terms of anastomotic leakage and 30-day mortality. In this study, which prospectively enrolled 925 patients in the LTG group and 1569 patients in the OTG group, although the LTG groups had significantly longer operating times, the LTG group had significantly shorter postoperative stays than the OTG group [35].

The extracorporeal anastomosis was performed in laparoscopic total gastrectomy to benefit from the experience gained in open surgery. Yan et al., in their study evaluating the results of minimally invasive EJ after laparoscopic or robotic total gastrectomy, found that intracorporeal EJ anastomosis had better results than extracorporeal EJ [23]. Anastomotic outcomes were influenced more by the anastomotic technique used and stapler preference than by whether the anastomosis was intracorporeal or extracorporeal. However, there is a lack of evidence on anastomotic technique and stapler preference, and surgeons use techniques based on their experience and training and are reluctant to use different techniques for EJ anastomosis, which carries a risk of serious morbidity.

A wide variety of intracorporeal anastomosis techniques have been developed and can be performed manually or with a stapler. Double stapling and hemi-double stapling techniques are techniques using double staplers without a need for purse-string suture and with ease of application [22,36,37,38]. In the double stapling technique (DST), the rod of the anvil is located at the centre of the linear staple line. In the HDST, it is located at the lateral edge. In DST and HDST, blood flow may be reduced at the intersection of linear and circular staple lines, resulting in anastomotic leakage. In addition, fibrosis at the crossing points may lead to anastomotic stenosis [13,38,39]. Yamauchi et al. [37] directly compared DST and HDST using OrVilTM through a randomised clinical trial. In their study, the intersection of the linear stapler and circular stapler, which was two locations in the DST group and one location in the HDST group, was reinforced in all cases with intracorporeal hand-sewn Lembert sutures. In the study, anastomosis times were significantly shortened with HDST. The authors did not find any superiority between DST and HDST in terms of postoperative complications in esophagojejunostomy after LTG in gastric cancer. However, they stated that HDST may be preferred in terms of the simplicity of the surgical technique.

Looking at the experience to date, we have seen that minimally invasive procedures have become widespread in the surgical treatment of stomach cancer, and laparoscopic methods have also found a place in guidelines. However, there is no consensus on which technique to use for esophagojejunostomy in operations where total gastrectomy is performed laparoscopically. SST, which is preferred in open surgery, has been difficult to perform in laparoscopy due to the narrow field of view and limited manoeuvrability. Placing the anvil in the oesophagus, cutting the oesophagus with a linear stapler and then performing the anastomosis with a circular stapler facilitated the procedure, as in the DST and HDST methods. Unfortunately, in these techniques, the linear stapler and the circular stapler overlap in two or one place. These intersections cause anastomotic stenosis and anastomotic leakage. To avoid this, the use of a purse-string suture and a single stapler for the anvil placed in the oesophagus is a very laborious method. The technique of performing a double stapler anastomosis without creating stapler overlap in the esophagogastrostomy has not been described until now. Based on this idea, we created a modified form of circular double stapling in end-to-side esophagojejunal anastomosis. There was no dog ear or overlapping of the stapler. In addition, anastomosis was created in an esophagus with a better blood supply. Another possible advantage of the technique is that the complete removal of the linear staple line provides a true proximal surgical margin rather than a doughnut. The mean proximal surgical margin was 2.6 ± 1.4 cm, and GDST is a suitable technique for maintaining adequate surgical margins in a patient population that includes tumours up to 1 cm above the Z line. 

Approximation of two lateral parts of the staple line to the anvil rod requires advanced surgical skills and additional time. The unavoidable disadvantage of this method is the longer total operative time and the longer time to perform esophagojejunal anastomosis in the GDST group. According to some studies [36,40], the reconstruction of the esophagojejunal anastomosis with SST requires more time than DST and HDST. Similarly, in the present study, surgical time is longer in GDST than in HDST due to performing manual sutures to approximate the whole linear staple line to the rod of anvil (292.6 ± 43.7 vs. 224.3 ± 36.1 min). Despite the technical difficulties and loss of operative time, it is clear that surgical modifications to reduce the complications associated with double stapling will continue to be explored, such as strengthening the anastomosis by placing a single layer of Lembert suture at the points of dog-ear formation and overlap, tightening the linear staple line with a purse-string device to prevent dog-ear formation, or suturing the seromuscular layer of the anastomosis continuously with a barbed suture, until the most appropriate technique emerges [38,41,42]. Technological developments in stapler anastomosis have not been very impressive for a long time. Bringing experts from different disciplines into the research field can pave the way for creative inventions. The esophagus and rectum, which are difficult anastomosis areas in gastrointestinal surgery, need such solutions the most. In these anastomoses, double stapler techniques will retain their place as the leading anastomotic technique in surgical studies due to their ease of use and safety. Overlapping staple lines in double stapler anastomoses remains a constant concern for surgeons. We have tried to completely eliminate this concern, which was partially reduced with HDST. We achieved this with the GDST method by first trapping the linear staple line of the esophagus into the circular stapler and then completely losing the linear staple line while creating an anastomosis by firing the circular stapler. By comparing patients who underwent GDST with patients who underwent HDST, we wanted to see if there was an advantage between the two methods, which have the same main purpose. Although we see that GDST is a feasible and safe method, we cannot make a strong recommendation for the technique due to the small number of patients. It is clear that randomised trials and more cases are needed to analyse the success of the method.

Recently, Aiolfi et al. from the University of Milan published a systematic review and meta-analysis of esophagojejunal anastomotic techniques [22]. In the meta-analysis, they noted that anastomosis-related complications may not reflect the quality of a particular technique and are influenced by surgeon skill, learning curves, and hospital volume. Anastomotic techniques evaluated in terms of anastomotic leakage and stenosis show similar risks. This systematic review included 20 studies, only one of which was a randomised trial. There was no significant difference between the anastomosis techniques in terms of anastomotic leakage. The number of patients in the groups was small in most of the included trials. However, the prevalence of anastomotic leakage was 3.6% for SST and 6.6% for HDST. We did not observe anastomotic leakage in our study. In the data of this review, the rate of anastomotic stenosis is higher in HDST than in SST, although there is no significant difference. We also had one case of anastomotic stenosis in the HDST group. No anastomotic stenosis was found in the GDST group. Since the GDST technique is the creation of an SST with DST modification, we believe that the SST results are useful for interpreting our technique. 

Our study had several limitations. The sample size of the groups was small, and only mid-term outcomes were available. Therefore, further studies with larger groups and longer follow-ups are needed. Another limitation is that the number of patients who received neoadjuvant therapy was significantly higher in the GDST group. Three of the four patients with peritoneal recurrence in the HDST group had received adjuvant chemoradiotherapy treatment, and a 79-year-old patient had received surgery only. All patients with locally advanced disease in the GDST group received neoadjuvant and adjuvant chemotherapy. The differences in treatment between the groups introduce a bias when considering the difference in survival.

## 5. Conclusions

We described a new technique for converting a double-stapled esophagojejunostomy to an actual single-stapled esophagojejunostomy. Esophagojejunostomy in laparoscopic total gastrectomy can be safely performed with both HDST and GDST. Further studies on GDST may be useful for laparoscopic esophagojejunostomy.

## Figures and Tables

**Table 1:** Patients and Tumour characteristics.

	HDST (*n* = 14)	GDST (*n* = 14)	*p*
Age (y), mean ± SD	58.0 ± 14.0	63.0 ± 8.0	0.174 ^c^
Men/women (*n*)	10/4	9/10	1.000 ^b^
BMI (kg/m^2^), mean ± SD	24.6 ± 5.6	29.3 ± 9.1	0.067 ^c^
ASA scores	*n* (%)	*n* (%)	
I	4 (28.6)	0 (0.0)	
II	5 (35.7)	3 (21.4)	0.035 ^b^
III	5 (35.7)	11 (78.6)	
Tumor localisation	*n* (%)	*n* (%)	
Upper–middle body of the stomach	9 (64.3)	7 (50.0)	
Siewert type II adenocarcinoma of the esophagogastric junction	3 (21.4)	4 (28.6)	0.775 ^b^
Siewert type III adenocarcinoma of the esophagogastric junction	2 (14.3)	3 (21.4)	
Lauren classification	*n* (%)	*n* (%)	
Intestinal type	9 (64.3)	11 (78.6)	
Diffuse type	3 (21.4)	3 (21.4)	0.565 ^b^
Mix type	2 (14.3)	0 (0.0)	
Neoadjuvant chemotherapy (*n*) (%)	5 (35.7)	10 (71.4)	0.023 ^a^
TNM classification *	*n* (%)	*n* (%)	
Stage I	1 (7.1)	2 (14.3)	
Stage II	1 (7.1)	4 (28.6)	0.347 ^b^
Stage III	12 (85.8)	8 (57.1)	
Retrieved lymph nodes (*n*), mean ± SD	23.1 ± 16.2	27.2 ± 6.2	0.234 ^c^
Lymph node metastasis (*n*), mean ± SD	9.5 ± 12.9	2.5 ± 3.4	0.069 ^c^
Distance from the surgical margin (cm) mean ± SD	2.5 ± 1.8	2.6 ± 1.4	0.725 ^c^

ASA: American Society of Anesthesiologists; HDST: hemidouble stapling technique; GDST: ghosting double stapling technique; *: American Joint Committee on Cancer TNM Staging Classification for Carcinoma of the Stomach 8th edition; ^a^: Pearson Chi-Square test; ^b^: Fisher’s Exact test; ^c^: Mann–Whitney U test; *p* < 0.05 statistically significant.

**Table 2:** Surgical details.

	HDST (*n* = 14)	GDST (*n* = 14)	*p*
Total operative time (min) mean ± SD	224.3 ± 36.1	292.6 ± 43.7	<0.001 ^b^
Time to perform EJ Anastomosis (min) mean ± SD	26.9 ± 6.2	38.6 ± 4.3	<0.001 ^b^
Blood loss (mL) mean ± SD	84.6 ± 41.6	82.5 ± 38.6	0.890 ^b^
Specimen removal			
Enlarging the trocar incision	11 (78.5)	14 (100.0)	
Suprapubic incision	2 (14.2)	0 (0.0)	0.186 ^a^
Transvaginal route	1 (7.3)	0 (0.0)	
Conversion to OTG	0 (0.0)	0 (0.0)	1.000 ^a^
Morbidity	2 (14.3)	3 (21.4)	1.000 ^a^

OTG: open total gastrectomy; HDST: hemi double stapling technique; GDST: ghosting hemi double stapling technique; EJ: esophagojejunal anastomosis; ^a^: Fisher’s Exact test; ^b^: Mann–Whitney U test; *p* < 0.05 statistically significant.

## Data Availability

This article includes all data generated or analysed during the study.
